# Catheter-directed thrombolysis guided by pulmonary artery pressure registration in pulmonary embolism: a case report

**DOI:** 10.1093/ehjcr/ytae015

**Published:** 2024-01-06

**Authors:** Ehssan Berenjkoub, Charlotte Kemper, Nicole Lewandowski, Marc Horlitz, Dennis Rottländer

**Affiliations:** Department of Cardiology, Krankenhaus Porz am Rhein, Urbacher Weg 19, 51149 Cologne, Germany; Department of Cardiology, Krankenhaus Porz am Rhein, Urbacher Weg 19, 51149 Cologne, Germany; Department of Cardiology, Krankenhaus Porz am Rhein, Urbacher Weg 19, 51149 Cologne, Germany; Department of Cardiology, Krankenhaus Porz am Rhein, Urbacher Weg 19, 51149 Cologne, Germany; Department of Cardiology, University Witten/Herdecke, Faculty of Health, School of Medicine, Alfred-Herrhausen-Str. 50, 58455 Witten, Germany; Department of Cardiology, Krankenhaus Porz am Rhein, Urbacher Weg 19, 51149 Cologne, Germany; Department of Cardiology, University Witten/Herdecke, Faculty of Health, School of Medicine, Alfred-Herrhausen-Str. 50, 58455 Witten, Germany

**Keywords:** Pulmonary embolism, Pulmonary artery pressure, Ultrasound-assisted catheter-directed thrombolysis, Case report

## Abstract

**Background:**

Duration and dosage of thrombolysis for ultrasound-assisted catheter-directed thrombolysis (UACDT) in patients with intermediate high-risk pulmonary embolism remain controversial and treatment protocols vary.

**Case summary:**

A 58-year-old female patient suffered from a right-sided urolithiasis. The clinical course was complicated by an intermediate high-risk pulmonary embolism [pulmonary embolism severity index (PESI) score 108 points and simplified PESI ≥1] with bilateral proximal thrombus and significant right heart dysfunction. The pulmonary embolism response team (PERT) made a decision towards UACDT. The standard duration of UACDT ranges between 6 and 15 h depending on clinical parameters. In this particular case, the clinical parameters such as heart rate (no tachycardia) or oxygen saturation (chronic obstructive pulmonary disease) might lead to premature termination of UACDT. Therefore, PERT decided to additionally monitor pulmonary artery pressure (PAP) continuously during the UACDT via a separate pigtail catheter in the pulmonary artery. Ultrasound-assisted catheter-directed thrombolysis was performed using 1 mg/h recombinant tissue plasminogen activator (rtPA) per catheter, while PAP was registered continuously. Heart rate and oxygen saturation remained unchanged during UACDT. However, after 6 h of UACDT, systolic PAP decreased slightly from 62 to 55 mmHg and therapy was prolonged to 15 h. Pulmonary artery pressure dropped to 46 mmHg after 15 h. The patient was discharged from hospital at Day 7, and echocardiography revealed no signs of right heart dysfunction.

**Discussion:**

Dosage of the thrombolysis agent and duration of UACDT are still a matter of debate. Besides clinical parameters and transthoracic echocardiography, invasive real-time PAP monitoring during UACDT could facilitate important information for therapy guidance in selected cases.

**Learning points**

- Treatment of pulmonary embolism should be guided by a local pulmonary embolism response team consisting of radiologist, cardiologist, angiologist, and intensive care medicine specialist.
- Ultrasound-assisted catheter-directed thrombolysis (UACDT) using the EkoSonic Endovascular System (EKOS™, Boston Scientific) is one therapeutic option in intermediate high-risk pulmonary embolism.
- Dosage of the thrombolysis agent and duration of UACDT are still a matter of debate. Besides clinical parameters and transthoracic echocardiography, invasive real-time pulmonary artery pressure monitoring during UACDT could facilitate important information for therapy guidance in selected cases.

## Introduction

In patients with intermediate-risk pulmonary embolism (PE), a systemic thrombolytic therapy prevented haemodynamic decompensation but increased the risk of major bleeding and stroke.^1^ Ultrasound-assisted catheter-directed thrombolysis (UACDT) is another therapeutic option for intermediate high-risk PE.^2^ It may also be considered in patients with high-risk PE in whom thrombolysis is contraindicated or has failed.^2,3^ Ultrasound-assisted catheter-directed thrombolysis is a targeted treatment and delivers the thrombolytic agent directly to the pulmonary artery (PA) in a reduced dosage compared with systemic thrombolysis, which might reduce the risk of major bleedings.^4^ Rapid reduction in clot size is achieved, facilitating quicker restoration of blood flow through the pulmonary vessels. This leads to early reduction in pulmonary artery pressure (PAP) and right ventricular (RV) afterload.^5–7^ Complications of PE, such as right heart failure and chronic thromboembolic pulmonary hypertension (CTEPH) might be addressed by this interventional therapy. However, inserting a PA catheter is an invasive procedure that carries risks, including infection, bleeding, and perforation. Furthermore, there is limited evidence supporting the use of UACDT, and the decision to employ this intervention should be based on careful consideration by the local PE response team (PERT).^2^ In case a decision towards UACDT in PE is made, careful patient monitoring is essential. Therefore, continuous PAP registration might increase procedural success and safety.

## Case summary

A 58-year-old Caucasian female patient suffered from a right-sided urolithiasis with hydronephrosis, which was treated by insertion of a ureteric stent. Despite deep vein thrombosis (DVT) stockings, no thrombosis prophylaxis agent was administered. However, the patient was transferred from the department of urology to the internal medicine ward due to dyspnoea (oxygen saturation 93% with 3 L oxygen per minute, heart rate 84/min, blood pressure 143/87 mmHg, and body mass index 30.0 kg/m^2^). The physical examination revealed cyanosis, jugular venous distention, and peripheral oedema. Crackles were detected upon lung auscultation, and an accentuated pulmonic component of the second heart sound was observed. The left calf exhibited tenderness, but otherwise the clinical examination of the lower limbs showed no significant findings. The medical history included type 2 diabetes, chronic obstructive pulmonary disease, and obesity. In 2013, a PE due to a left-sided DVT was treated by warfarin for 6 months. The aetiology of PE was attributed to immobilization during a long-distance flight.

An arterial blood gas analysis (BGA) showed a respiratory alkalosis due to hyperventilation (respiratory rate 30/min) and hypoxemia, despite oxygen therapy (pH 7.52, pO_2_ 59 mmHg, pCO_2_ 28 mmHg, SpO_2_ 93.5% BE 1.1; 3 L oxygen). The baseline haemoglobin, platelet count, international normalized ratio, and partial thromboplastin time (PTT) were within normal ranges (12.2 g/dL, 161/nL, 1.62, and 27 s). Electrocardiogram (ECG) revealed right bundle branch block and d-dimer (4.0 mg/L, reference range < 0.5 mg/L) were elevated. Transthoracic echocardiography (TTE) showed normal left ventricular (LV) function. However, significant RV dysfunction, a RV/LV ratio of 1.04, and normal RV wall diameter (3 mm) were observed. McConnell’s sign, pulsed wave notching of the PA, and LV D-shape were present. Furthermore, PAP and tricuspid regurgitation velocity were significantly elevated (68 mmHg and 3.45 m/s). Doppler ultrasound revealed left-sided popliteal DVT.

**Figure 1:**
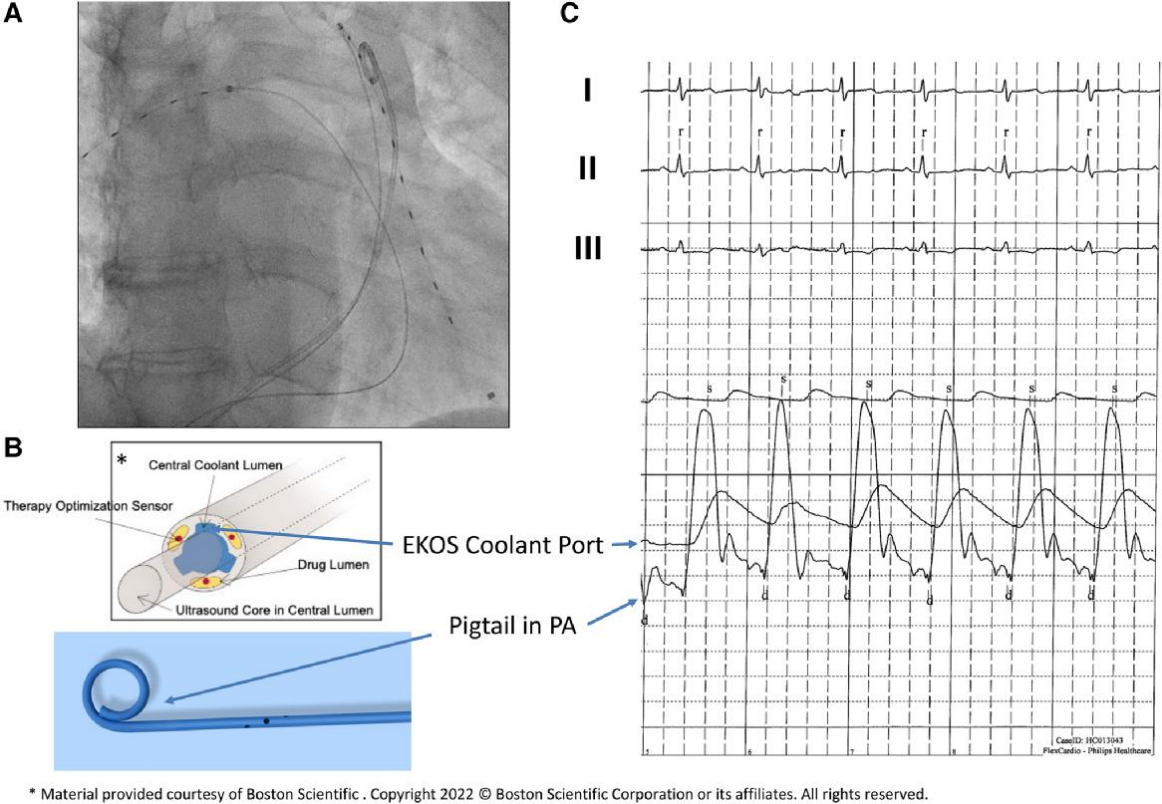
Real-time monitoring of pulmonary artery pressure using a pigtail catheter. (*A*) Fluoroscopy with ultrasound-assisted catheter-directed thrombolysis catheter in the left and right pulmonary arteries. Pigtail catheter in the trunk of the pulmonary artery. (*B*, *C*) Pulmonary artery pressure registration via the EKOS Coolant Port and the pigtail catheter as indicated by arrows.

**Figure 2:**
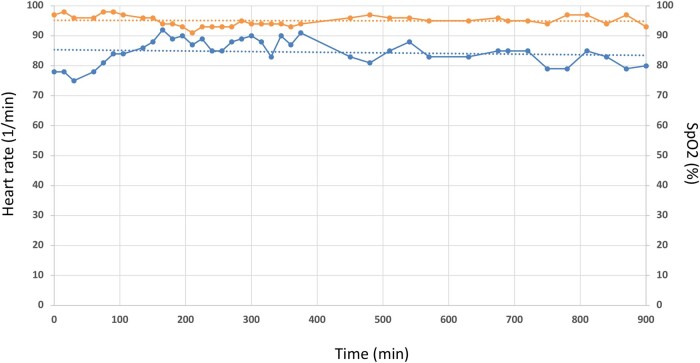
Monitoring of heart frequency and SpO_2_ during ultrasound-assisted catheter-directed thrombolysis. Heart rate in 1/min (lower curve) and SpO_2_ in % (upper curve) during 900 min of ultrasound-assisted catheter-directed thrombolysis. Dotted lines indicate trend lines.

**Figure 3:**
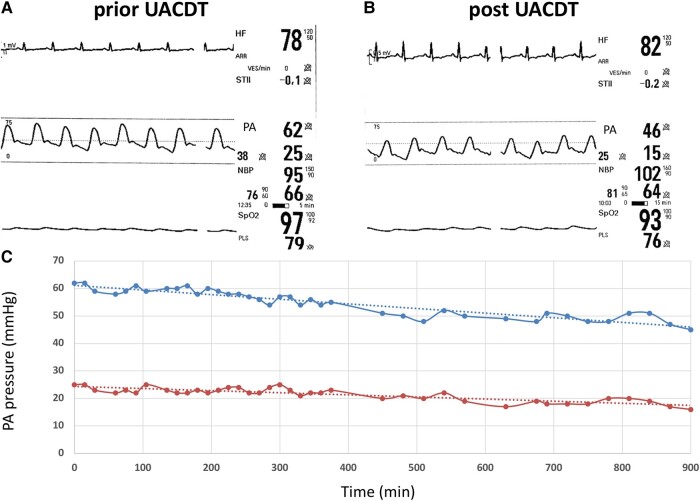
Real-time pulmonary artery pressure monitoring during ultrasound-assisted catheter-directed thrombolysis. Monitor snapshots prior (*A*) and post (*B*) ultrasound-assisted catheter-directed thrombolysis showing electrocardiogram, heart rate, real-time pulmonary artery pressure measured via pigtail in the pulmonary artery, non-invasive blood pressure, and SpO_2_. (*C*) Systolic pulmonary artery pressure (upper curve) and diastolic pulmonary artery pressure (lower curve) as a function of time (min). Dotted lines indicate trend lines. UACDT, ultrasound-assisted catheter-directed thrombolysis; PA, pulmonary artery.

**Figure 4:**
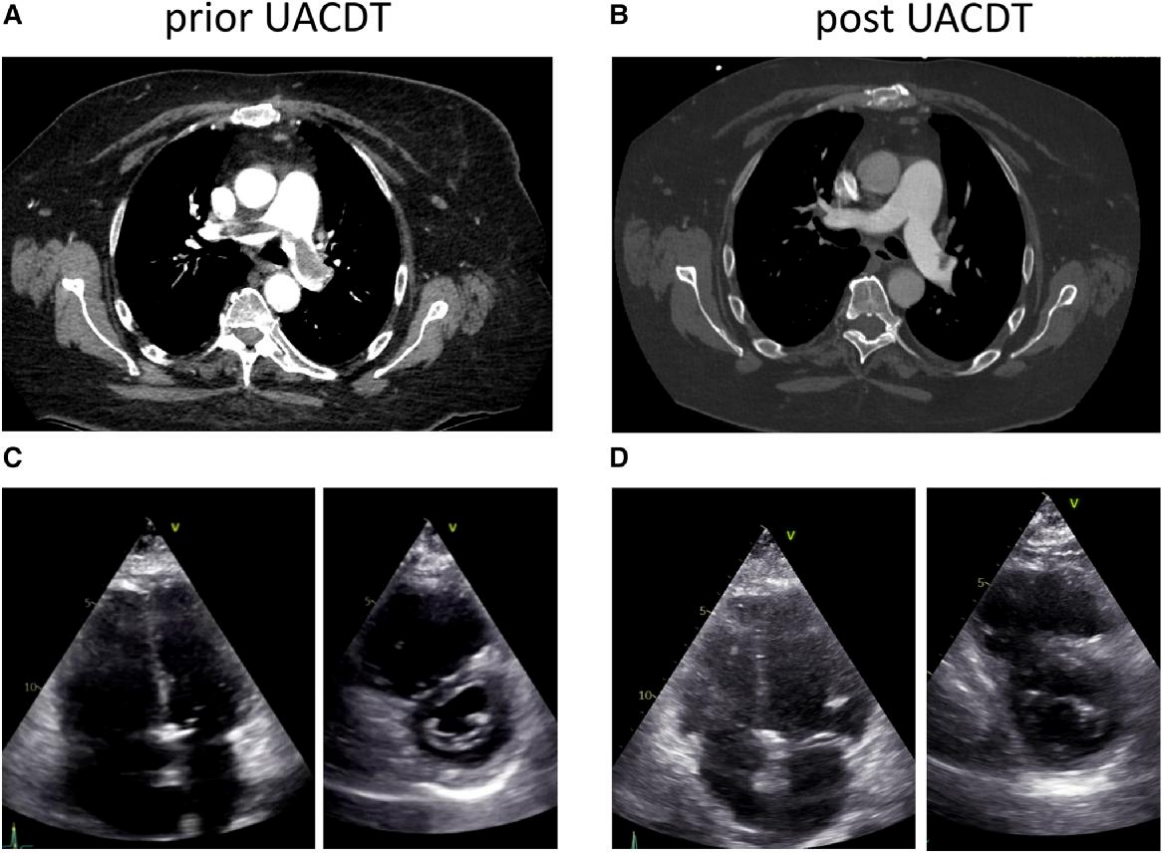
Computed tomography pulmonary angiography and transthoracic echocardiography prior and post ultrasound-assisted catheter-directed thrombolysis. Computed tomography pulmonary angiography prior (*A*) and post (*B*) ultrasound-assisted catheter-directed thrombolysis indicating nearly complete resolution of pulmonary thrombus. Transthoracic echocardiography prior (*C*) and post (*D*) ultrasound-assisted catheter-directed thrombolysis indicating resolving signs of right heart dysfunction. UACDT, ultrasound-assisted catheter-directed thrombolysis.

A computed tomography (CT) pulmonary angiography (CTPA) confirmed a PE with bilateral proximal thrombus and showed no signs of CTEPH (see Supplementary material online, *Video S1*). High sensitive troponin T (47 pg/mL, reference range < 14 pg/mL) and NT-proBNP (30 939 pg/mL, reference range < 900 pg/mL) were elevated. Local PERT reviewed the case. The PE severity index (PESI) was calculated at 108 points (intermediate high-risk, class IV; chronic lung disease, respiratory rate ≥ 30, arterial oxygen saturation < 90% without oxygen), and simplified PESI (sPESI) was ≥1. Intravenous anticoagulation with heparin (bolus 5000 IE, weight adjusted to 1600 units per hour) was started, and the patient transferred to the intermediate care unit. After 24 h of heparin administration (PTT repetitive > 80 ms), the patient remained symptomatic with severe RV dysfunction. Based on a consensus statement, the PERT made a decision towards UACDT, while UACDT is not recommended following the current European society of cardiology guidelines.^2,3^ After discussing the risks (bleeding, infection, and perforation) and benefits (rapid clot resolution, reduction RV dysfunction, and probable prevention of CTEPH) of UACDT and considering alternative therapeutic approaches, the patient consented to UACDT therapy, especially given his history of PE. The EkoSonic Endovascular System (EKOS™, Boston Scientific) uses high-frequency, low-power ultrasound energy combined with a low-dose thrombolytic agent to dissolve the pulmonary thrombus. However, in this case, clinical parameters such as heart rate (no tachycardia) or oxygen saturation (chronic obstructive pulmonary disease) might lead to premature termination of UACDT. Furthermore, TTE was limited by imaging quality (obesity and impaired insonation angle). Therefore, PERT decided to additionally monitor PAP continuously during the UACDT via a separate transfemoral pigtail catheter in the PA. EKOS™ catheters were placed into both PAs via a transfemoral venous access. Furthermore, using a third transfemoral access, a pigtail catheter was positioned in the trunk of the PA. Ultrasound-guided micropuncture was used to reduce the bleeding risk. Of note, PAP registration via the cooling port of the EKOS™ catheter showed dampened pressure curves (*Figure 1*). Ultrasound-assisted catheter-directed thrombolysis with a standard 6 h low-dose recombinant tissue plasminogen activator (rtPA) protocol (OPTALYSE Group 3) was used (1 mg/h rtPA per catheter, 12 mg in total). Heart rate was normal during the procedure and SpO_2_ remained unchanged (*Figure 2*). After 6 h of UACDT, the clinical status of the patient was checked and TTE performed. Pulmonary artery pressure decreased from 62 to 55 mmHg, which led to the decision to prolong the therapy to 15 h (0.5 mg/h rtPA per catheter for another 9 h, 21 mg in 15 h total rtPA dose). Pulmonary artery pressure further dropped to 46 mmHg after 15 h (*Figure 3*). The patient was transferred 8 h after UACDT to the cardiology ward (no oxygen demand, heart rate 68/min, SpO_2_: 97%). Transthoracic echocardiography showed resolving signs of RV dysfunction (*Figure 4* and *Table 1*). Heparin was replaced by rivaroxaban 15 mg twice daily 6 h following UACDT (4 h after transfemoral sheath removal). *Figure 4* shows the results of CTPA and TTE prior and post UACDT. Patient was discharged from hospital on Day 7. Transthoracic echocardiography revealed no signs of RV dysfunction (PAP 38 mmHg).

**Table 1:** Transthoracic echocardiography at baseline, post ultrasound-assisted catheter-directed thrombolysis, and at discharge

	Baseline	Post USAT	Discharge (Day 7)
RV/LV ratio	1.04	0.95	0.88
sPAP (mmHg)	68	43	38
D-Shape	+	+	−
McConnell	+	+	−
TAPSE (mm)	18	19	26
LVEDD basal (cm)	4.6	4.7	4.8
RVEDD basal (cm)	4.8	4.5	4.2
VCI diameter (mm)	25	19	21
RA area (cm^2^)	23	23	20
S′	11	12	13
PV acceleration time (ms)	54	58	83
PV notching	+	+	−

RV, right ventricle; LV, left ventricle; sPAP, systolic pulmonary artery pressure; LVEDD, left ventricular end-diastolic diameter; RVEDD, right ventricular end-diastolic diameter; VCI, vena cava inferior; RA, right atrium; PV, pulmonary valve.

Three months after UACDT, a follow-up in the outpatient clinic was scheduled. Transthoracic echocardiography showed normalized PAP and no signs of RV dysfunction. The patient reported of no dyspnoea.

## Discussion

Pulmonary embolism is a major cause of cardiovascular death in Europe.^8^ Intermediate high-risk PE is associated with increased mortality and complications such as right heart failure and CTEPH.^9^ These patients exhibit signs of RV dysfunction on echocardiography, coupled with elevated troponin levels indicating myocardial injury. In most cases, therapeutic anticoagulation alone is sufficient and UACDT not needed. Nonetheless, some patients may experience worsening conditions due to progressive RV failure. About 5% of initially anticoagulated patients face haemodynamic decompensation, primarily within the initial 72 h after admission, necessitating rescue reperfusion treatment.^1^ Early mortality may range from 5 to 10% in initially normotensive patients with right ventricular dysfunction and myocardial injury.^10^ It is necessary to closely monitor initially normotensive patients at an elevated risk of haemodynamic deterioration. To date, UACDT is not recommended routinely in patients with intermediate high-risk PE.^2,3^ This recommendation is based on the limited clinical data and missing randomized controlled trials.^3^ In patients with intermediate high-risk PE and lack of improvement on anticoagulation, UACDT might be considered after an individual decision by PERT. Ultrasound-assisted catheter-directed thrombolysis is associated with improvements in RV function and lung perfusion.^4–7^ It revealed in randomized clinical trials reduced incidence of pulmonary hypertension, lower PAP, improved RV dysfunction, and lower mortality in patients with intermediate high-risk PE.^4–7^

The OPTALYSE trial was a randomized trial in patients with intermediate high-risk PE, which showed EKOS™ therapy to be safe and effective with low dosages of thrombolysis and short-duration protocols.^7^ However, one might speculate that an individual therapy regime for each patient might facilitate the highest clinical success and lowest peri-interventional bleeding rate. Clinical parameters such as heart rate (e.g. atrial fibrillation and beta-blocker therapy) or oxygen saturation (pre-existing chronic obstructive pulmonary disease) might lead to premature termination of UACDT. Repetitive TTE to determine PAP and RV dysfunction under UACDT therapy is recommended and important for patient monitoring and decision-making. However, sometimes, TTE is limited by imaging quality. Invasive continuous registration of PAP might overcome these limitations and deliver real-time data on procedural success.

This is the first case report of continuous PAP monitoring in UACDT. This approach exhibited distinct advantages over repetitive TTE and clinical parameters:

1. Direct identification of PAP decrease
2. Independence from image quality
3. Valuable decision support for therapy prolongation
4. Access to central venous oxygen saturation
5. Complement to clinical and echocardiographic parameters
6. Individualized therapy protocols to reduce rtPA dose and UACDT time

Therefore, continuous PAP monitoring could emerge as a tool to enhance the procedural outcome of UACDT.

However, there are limitations associated with continuous PAP monitoring during UACDT, such as the additional femoral puncture (increasing the risk of bleeding), catheter dislodgement, or dampened pressure curves leading to data misinterpretation. Additionally, relying solely on clinical parameters might be sufficient to guide UACDT, and clear PAP cut-off values for therapy success are missing. Considering pressure registration, a Swan–Ganz catheter might be an alternative. Nonetheless, a pigtail catheter was chosen due to its atraumatic nature, minimal dampening of pressure registration, reduced thrombogenicity owing to a smaller surface, and the availability of central venous blood gas analyses. Jugular catheter insertion could be an alternative to mitigate bleeding complications. However, we assumed that using ultrasound-guided femoral micropuncture for the placement of UACDT catheters and the pigtail catheter would be feasible. In summary, further clinical trials are needed to elucidate the role of continuous PAP registration in UACDT.

## Conclusion

Continuous PAP monitoring using a dedicated transfemoral pigtail catheter is feasible and might help to identify the individual rtPA dosage and UACDT duration in selected cases.

## Lead author biography


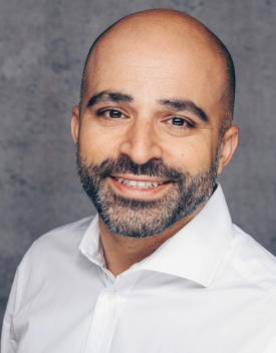
University of Cologne Medical School from 1999 to 2005 followed by a licence to practice medicine in Germany (MD). Resident in cardiology at the Hospital Cologne Porz from 2005 to 2013 completed by board certification in cardiology. Afterwards, consultant in interventional cardiology with a special focus on device therapy.

## Supplementary Material

ytae015_Supplementary_Data

## Data Availability

The data underlying this article will be shared upon reasonable request to the corresponding author.

## Supplementary material

**Consent:** According to the COPE guidelines, the patient has given his written consent for submission and publication of this case report (including images and associated text).

**Funding:** None declared.
